# Three-dimensional ultrasound for knee osteophyte depiction: a comparative study to computed tomography

**DOI:** 10.1007/s11548-021-02456-4

**Published:** 2021-07-27

**Authors:** Valeria Vendries, Tamas Ungi, Jordan Harry, Manuela Kunz, Jana Podlipská, Les MacKenzie, Gabriel Venne

**Affiliations:** 1 Anatomical Sciences Program and Department of Biomedical and Molecular Sciences, Queens University, Kingston, ON K7L3 N6 Canada; 2 School of Computing, Queen’s University, Kingston, ON K7L 3N6 Canada; 3 Research Unit of Medical Imaging, Physics and Technology, Faculty of Medicine, University of Oulu, Oulu, Finland; 4 Department of Anatomy and Cell Biology, McGill University, Montreal, QC H3A 0G4 Canada

**Keywords:** 3D ultrasound, Computed tomography, Structured light scanner, Osteophyte, Osteoarthritis

## Abstract

**Purpose:**

Osteophytes are common radiographic markers of osteoarthritis. However, they are not accurately depicted using conventional imaging, thus hampering surgical interventions that rely on pre-operative images. Studies have shown that ultrasound (US) is promising at detecting osteophytes and monitoring the progression of osteoarthritis. Furthermore, three-dimensional (3D) ultrasound reconstructions may offer a means to quantify osteophytes. The purpose of this study was to compare the accuracy of osteophyte depiction in the knee joint between 3D US and conventional computed tomography (CT).

**Methods:**

Eleven human cadaveric knees were pre-screened for the presence of osteophytes. Three osteoarthritic knees were selected, and then, 3D US and CT images were obtained, segmented, and digitally reconstructed in 3D. After dissection, high-resolution structured light scanner (SLS) images of the joint surfaces were obtained. Surface matching and root mean square (RMS) error analyses of surface distances were performed to assess the accuracy of each modality in capturing osteophytes. The RMS errors were compared between 3D US, CT and SLS models.

**Results:**

Average RMS error comparisons for 3D US versus SLS and CT versus SLS models were 0.87 mm ± 0.33 mm (average ± standard deviation) and 0.95 mm ± 0.32 mm, respectively. No statistical difference was found between 3D US and CT. Comparative observations of imaging modalities suggested that 3D US better depicted osteophytes with cartilage and fibrocartilage tissue characteristics compared to CT.

**Conclusion:**

Using 3D US can improve the depiction of osteophytes with a cartilaginous portion compared to CT. It can also provide useful information about the presence and extent of osteophytes. Whilst algorithm improvements for automatic segmentation and registration of US are needed to provide a more robust investigation of osteophyte depiction accuracy, this investigation puts forward the potential application for 3D US in routine diagnostic evaluations and pre-operative planning of osteoarthritis.

## Introduction

One of the main challenges in assessing the severity of knee osteoarthritis lies in the accurate depiction of bone and cartilage abnormalities by medical imaging modalities [1, 2]. For instance, osteophyte formation—abnormal osseocartilaginous outgrowths usually located at bone margins—which are a major criterion in osteoarthritis diagnostic [3, 4], is not always clearly depicted using conventional imaging. This may hamper the accuracy of early diagnosis and of pre-operative evaluation for subsequent surgical interventions. Their variability in density, composition and location within the joint may contribute to the reasons why they are not well depicted using conventional radiographs (CR) [5–7]. Prior research has suggested that osteophytes may not be accurately depicted on conventional pre-operative computed tomography (CT) images, particularly for osteoarthritic joints prior to joint replacement surgery [5]. Such a suggestion may therefore represent one possible reason for the unforeseen encounter of osteophytes that contribute to intraoperative alignment errors of the surgical instrumentation [8]. Furthermore, Al-attar et al. encountered CT to be optimal for the representation of more calcified type of osteophytes but inaccurate in depicting immature osteophytes [9].

Previous studies have investigated osteophyte detection of using US compared to other modalities and shown that US is a promising tool to detect articular changes such as early-stage osteophytes [6, 10–15]. For instance, Koski et al. 2016 developed a novel atlas for semi-quantitatively scoring the severity of osteophytes in the tibiofemoral (knee) joint using US [12]. Their study demonstrated that US was more sensitive than CR at depicting osteophytes which was later confirmed by Podlipská et al. 2016 in a study investigating the diagnostic performance of US using magnetic resonance imaging (MRI) as a reference tool [11]. The relevance of these findings is such that osteophyte size as revealed by US can be used as a predictor of degenerative changes occurring in osteoarthritic joints [12].

Whilst some studies have evaluated osteophyte number, size and dimensions using US [6, 12, 16, 17], to date and to the authors’ knowledge, there are no studies reporting surface and volume quantification of osteophytes using US.

This preliminary study puts forward a method to quantify the accuracy of osteophyte depiction from 3D reconstructions of tracked US compared to CT. 3D reconstructed US, in this study referred to as “3D US”, combines the accessibility of US with the ability to reconstruct the images similarly to CT or MRI [18]. Furthermore, it involves no ionizing radiation and is becoming a modality of interest in the fields of joint imaging and orthopaedic surgery [19]. This can be particularly useful in the field of computer-assisted orthopaedic surgery (CAOS), given that osteophytes are not well depicted using conventional modalities interfering with the generation of accurate models for image-dependent interventions and with the surgical procedure itself [20, 21].

Since there is a need to obtain comprehensive surface representation from three-dimensional imaging at the sites of osteophytes including bony and cartilaginous areas, the principal objective of the present study was to study how 3D reconstructed US and CT depict osteophytes compared to a ground truth model of the joint surface captured using structured light scanning (SLS).

## Materials and methods

**Fig. 1:**
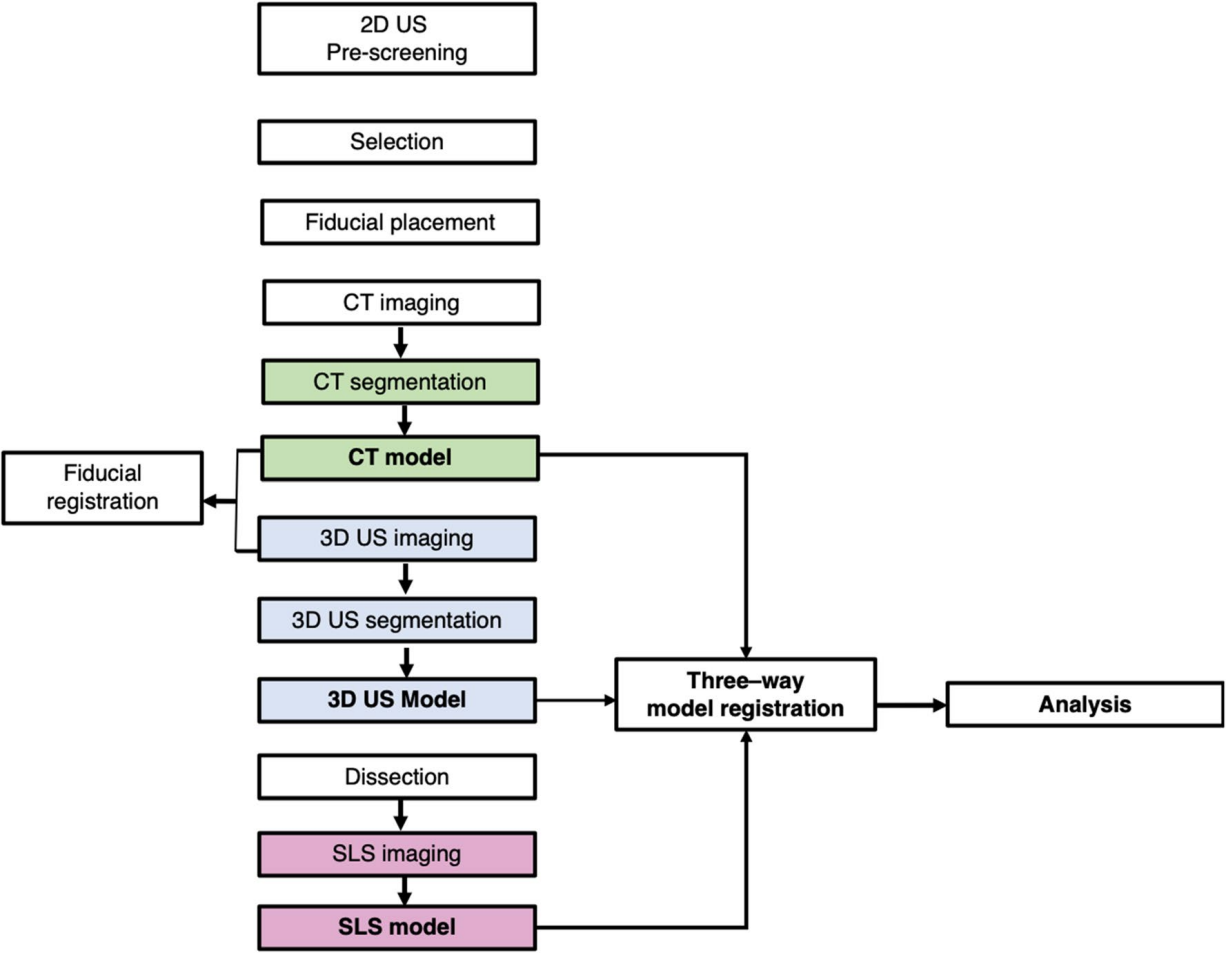
Overview of the study methodology

Eleven fresh frozen–thawed human cadaveric knees were obtained from the Queen’s University Body Donation program. Knees were pre-screened for the presence of osteophytes, followed by image acquisition for the generation of 3D models. Comparison between imaging modalities was performed at last. Figure 1 illustrates the study methodology.

### Pre-screening with US

**Fig. 2:**
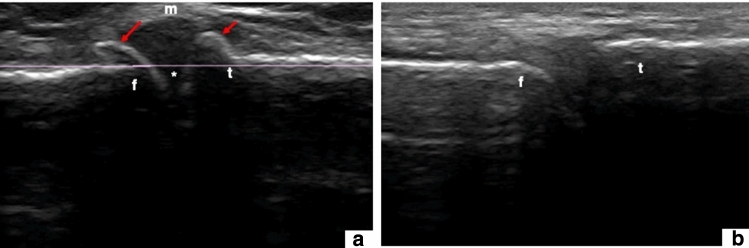
2D US pre-screening evaluation. **a** A representative 2D US of a knee side included in the study with osteophytes (*red arrows*), possibly joint space narrowing (*) and meniscal extrusion (m). **b** A representative 2D US of a knee side showing no osteophytes. f = femur; t = tibia

The US probe (L12- 5L40S-3 linear probe, MicrUS Ext-1H Digital Ultrasound system, TELEMED Ltd, Vilnius, Lithuania, EU) was used to sweep over the medial and lateral sides of the knees, positioned supine and in full extension. All knees were pre-screened according to the previously validated US semi-quantitative grading system described by Koski et al. [12], (Table 1). It has been suggested that semi-quantitative US atlases can be helpful as references by providing well-defined examples of the presence of osteophytes during research and clinical contexts [12, 13]. Any abnormal step-up prominence from the bone margin (between 1 and 3) together with other visible signs of osteoarthritis was considered as inclusion criteria. Initial 2D US evaluation revealed possibility of osteoarthritis in six out of eleven specimens (specimens 1–6). Selected knees were resected from the donor and refrozen until the imaging protocol. Figure 2 includes examples of a knee included for the study (a) and an excluded knee (b).

**Table 1:** Atlas-based knee osteophyte assessment used as inclusion/exclusion criteria for the selection of osteoarthritic knees based on the presence of osteophytes

Ultrasound grade^a^	
0	No osteophyte
1	Small osteophyte
2	Medium osteophyte
3	Large osteophyte

^a^The extent of osteophyte size is described according to the US-based semi-quantitative grading system described by Koski et al. [12]

### Imaging protocol and 3D model generation

Prior to imaging, selected knees were thawed to room temperature. Four screws were screwed into the femur as fiducial for image registration purposes. 3D US and CT scans were obtained and segmented to digitally reconstruct 3D knee models. Knees were dissected to expose the distal femur, where the presence of osteophytes was confirmed on three out of the six specimens, out of which a total of 5 femoral knee sides (the medial side of specimens 1–3, and the lateral sides of specimens 2 and 3) were selected for surface scanning using SLS. The surface topography of the exposed anatomy captured with SLS served as ground truth model. From each femoral side, osteophyte areas were circumscribed for a total of *n* = 18 osteophyte regions evaluated. Such areas of osteophytes in their corresponding virtual models (3D US, CT and SLS) were subjected to image modality comparison.

#### Computed tomography

CT images were obtained (GE BrightSpeed 16-slice scanner; 0.625 mm slice thickness, and voltage exposure of.120 kV); pixel size for all scans was on average 0.36 × 0.36 mm, with a minimum size of 0.35 × 0.35 mm and a maximum size of 0.39 × 0.39 mm. Segmentation and virtual 3D surface models from the distal femur were created using a commercial package (Mimics software, Materialise, Leuven, Belgium, version 15.0 or later). Initial automatic segmentation was performed selecting a threshold of pixels higher than 226 Hounsfield units (HU), which corresponds to bone tissue density and further edited to isolate the distal femur. This was manually refined in all three planes to create more accurate representations of the anatomy of interest.

#### 3D US (reconstruction of tracked US)

US images, fiducial registration and electromagnetic tracking data were acquired and spatially 3D-reconstructed using the open-source 3D Slicer (www.slicer.org, with SlicerIGT (www.slicerigt.org) [22] extension with the embedded PLUS toolkit (www.plustoolkit.org) [23]). Fiducial points were collected from the anatomy, to register the recorded US images to the 3D CT models. US images were obtained (SonixTablet ultrasound machine, Ultrasonix, Medical Corp, Richmond, BC, Canada) from the medial and lateral sides of the knee joint using a L14/38 linear transducer at a frequency of 14 MHz and an imaging depth of 4 cm. The gain and other US parameters were adjusted in order to maximize the quality of the images. Stylus for localizing fiducial points and real-time tracking of US images were done using an NDI Ascension TrakSTAR (Waterloo, ON, Canada) electromagnetic tracker. 3D Slicer was used to manually segment the reconstructed 3D US volumes from which virtual 3D surface models of the medial and lateral sides of the distal femur were created. Voxel size in all three dimensions was chosen at 0.5 mm resolution in the volume reconstruction.

#### Structured light scanning

**Fig. 3:**
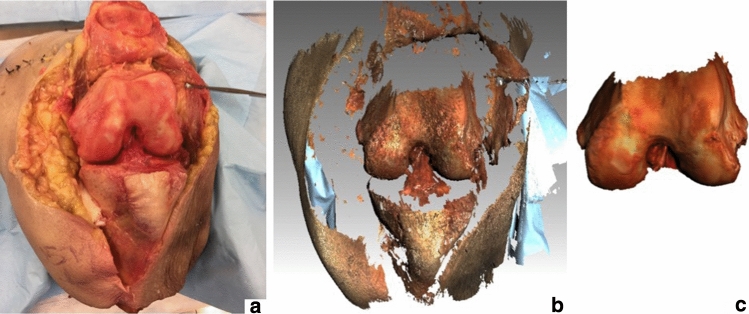
Knee dissection and surface light scan (SLS) model generation. **a** Dissected knee; **b** acquired SLS of the exposed anatomy; **c** final SLS model of the femur after post-processing

SLS from the knees with osteophytes was captured using the Artec Spider (Artec Group, Luxembourg) hand-held structured light scanner, with a reported 0.1 mm 3D resolution, and a 0.05 mm 3D point accuracy. Scans were post-processed, and 3D models were generated using Artec studio 11 software as in Figure 3.

### Image modality comparison for osteophyte depiction

#### Three-Way Model registration

The three data sets (CT, 3D US, SLS) were aligned in the same coordinate system by means of rigid registration, and this was performed using an iterative closest point (ICP) algorithm [24]. The CT model displayed the largest portion of the anatomy; therefore, the SLS and 3D US models were registered to the CT coordinate system chosen as the reference.

Registration of the SLS models to the CT models was initiated by global registration using anatomical landmarks and refined using the ICP algorithm. For this, the registered SLS model was cropped to contain only bony surfaces and the ICP algorithm was applied again using only the bony surfaces between the SLS model and the CT model. The result of the refined registration was then applied to the complete SLS model containing bone and articular cartilage information.

An initial fiducial-based registration between the US volumes and the CT models was previously achieved during 3D US scanning. The 3D US models of the medial and lateral sides of the distal femur were combined into one complete model. This complete model was cropped in 3D Slicer to contain only bony surfaces, and the ICP algorithm was applied using only the bony surfaces between the SLS model (already aligned with CT) and the 3D US model. The result of the refined registration was then applied to the complete 3D US model containing bone and articular cartilage information. Errors for the registration between the bony surfaces of the SLS model, the 3D US model and the CT model were reported as root mean square (RMS) errors.

#### Surface matching analysis and topographic map generation

A previously used custom software [5, 9] was utilized to create a surface match and calculate the RMS between the CT or 3D US models and the SLS models of each of the osteophytic regions. RMS distance between surfaces was obtained as a quantitative measurement of the distance error between the SLS model and the CT model or the 3D US model. Additionally, colour-coded topographic maps were generated to visually represent the magnitude of the distance between the compared models.

#### Statistical analysis

Statistical analysis was conducted using paired t-tests for unequal distribution in MATLAB and Statistics Toolbox Release (2014, version 8.3) (The MathWorks, Inc., Massachusetts, USA), with significance at p ≤ 0.05.

## Results

The RMS registration errors for the alignment between the SLS and 3D US models to the CT model are of submillimetre magnitude as indicated in Table 2.

**Table 2:** Global surface registration error between the surface models acquired for each specimen

RMS registration errors (mm)		
Specimen	SLS-CT^a^	3D US-CT^b^
Specimen 1	0.96	0.70
Specimen 2	0.58	0.59
Specimen 3	0.94	0.62

^a^SLS-CT: represents the alignment of the SLS to the CT coordinate system.

^b^3D US-CT: represents the alignment of the 3D US to the CT coordinate system

### 3D US versus CT osteophyte depiction analysis

Analysis of RMS surface distances for the CT to SLS (CT-SLS) and the 3D US to SLS (3D US-CT) comparisons of all 18 osteophyte regions, showed no statistical difference (Table 3).

**Table 3:** RMS surface distances for the total number of osteophyte regions evaluated (*n* = 18)

RMS surface distance (mm)			
Specimen	Osteophyte regions	CT-SLS^a^	3D US-SLS^b^
Specimen1M (*n* = 4)	o1	1.30	0.56
	o2	1.30	0.51
	o3	1.37	0.61
	o4	1.03	0.83
Specimen2M (*n* = 3)	o1	1.30	1.45
	o2	1.18	1.16
	o3	1.20	1.34
Specimen 2L (*n* = 5)	o1	0.53	0.67
	o2	0.43	0.88
	o3	0.42	0.42
	o4	0.79	0.57
	o5	1.04	0.75
Specimen 3L (*n* = 3)	o1	0.63	0.63
	o2	0.92	1.25
	o3	1.25	1.48
Specimen 3 M (*n* = 3)	o1	0.57	0.94
	o2	0.82	0.75
	o3	0.97	0.88
Average		0.95	0.87
SD		0.32	0.33

^a^CT-SLS: comparison between the CT and the SLS models; ^b^3D US-SLS: comparison between the 3D US and the SLS models.

M medial, L lateral; *n* = No. of osteophyte regions. SD  standard deviation

**Fig. 4:**
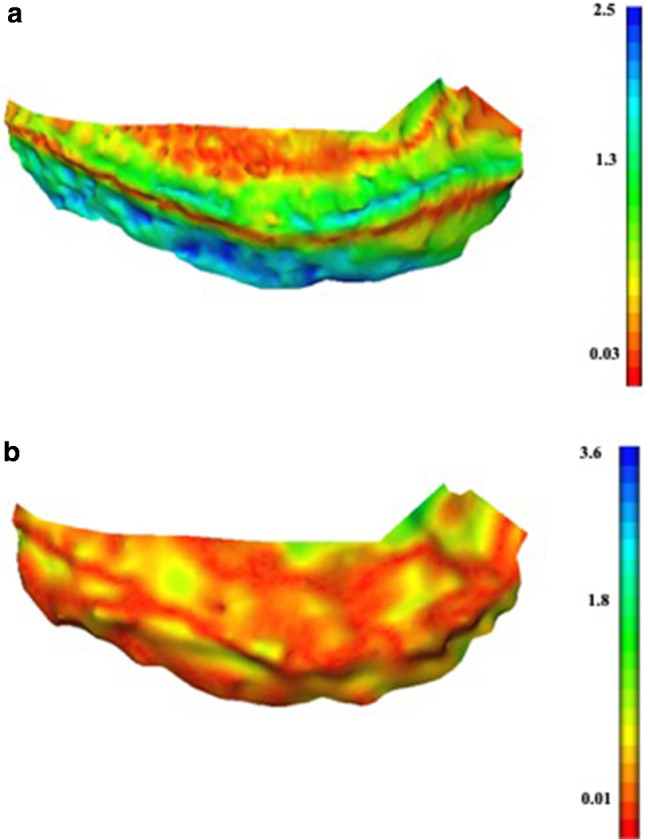
Colour maps of the medial side of specimen 1 showing a more accurate osteophyte depiction in 3D US. **a** Absolute error colour map for the CT-SLS comparison; **b** absolute error colour map for the 3D US-SLS comparison

**Fig. 5:**
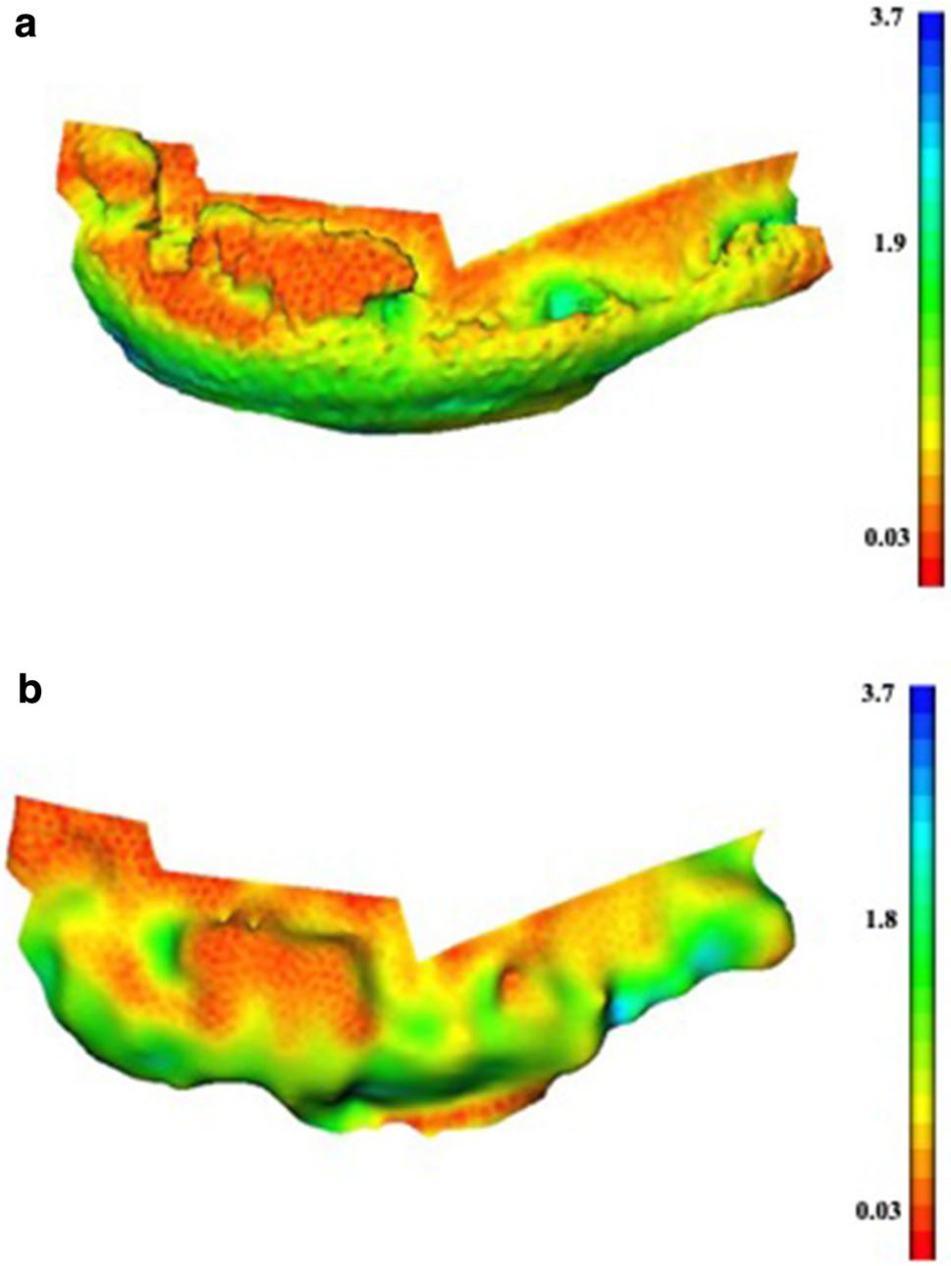
Colour maps for a case of similar osteophyte depiction between 3D US and CT. Representative images are shown for the medial side of specimen 2. **a** Absolute error colour map for the CT-SLS comparison; **b** absolute error colour map for the 3D US-SLS comparison

Average osteophyte RMS surface distances per specimen are highlighted in Table 4. The medial side of specimen 1 showed that the RMS surface distance average for 3D US-SLS (0.63 mm) was significantly lower than that of the CT-SLS (1.25 mm) comparison. Higher distances were observed between the CT-SLS comparison at the edges of osteophytes and at the articular surface, as represented by green–blue-toned values on the coloured map (Fig. 4a). In contrast, 3D US revealed a more even colour distribution with lower RMS surface distances as represented by yellow–red-toned values (Fig. 4b). RMS surface distance averages for specimens 2 and 3 were not statistically different. The coloured maps of the osteophyte areas illustrated a similar and evenly spread colour distribution (Fig. 5).

**Table 4:** Surface distances for within-specimen osteophyte evaluation

Average RMS surface distances (mm) per specimen						
		CT-SLS^a^	3D US-SLS ^b^			
Specimen	No. of osteophyte regions	Average	SD	Average	SD	
Specimen 1 M	4	1.25	0.15	0.63	0.14	*p* = 0.001
Specimen 2 M	3	1.23	0.06	1.32	0.15	
Specimen 2L	5	0.64	0.27	0.66	0.17	
Specimen 3 M	3	0.79	0.20	0.86	0.10	
Specimen 3L	3	0.93	0.31	1.12	0.44	

^a^CT-SLS: comparison between the CT and the SLS models

^b^3D US-SLS: comparison between the 3D US and the SLS models.

M  medium, L lateral, *SD*  standard deviation; *p* = *p*-value

## Discussion

The inaccurate imaging of osteophytes represents not only problems for monitoring the progression of this prevalent disease, but also interferes with the design of image-dependent surgical interventions and CAOS [8]. It has been suggested that US can reliably depict osteophytes in the knee joint [6, 10–12, 17]. Results from the present study support such previous observations. This pilot study developed a research protocol for quantifying the differences in knee osteophyte depiction compared to virtual 3D models generated from the segmentation of 3D US and CT volumetric data sets at the sites of osteophytes to a ground truth surface model, the SLS. Importantly, the present results show similar osteophyte depiction ability using 3D US compared to CT; nonetheless, in some specimens' 3D US was observed to be potentially better at depicting cartilaginous regions.

Comparison between 3D US and CT across all osteophytic regions suggested that there was no statistical difference between the two modalities at depicting osteophytes. Average RMS surface distance for 3D US-SLS comparison was similar to that of the CT-SLS. Similarity in the RMS surface distances to the SLS suggested that both modalities may be comparably capable of osteophyte depiction.

Osteophyte depiction was evaluated for each individual specimen. 3D US showed to be more accurate for osteophyte depiction compared to CT in the medial side of specimen 1. Bony areas appeared to align properly and with minimal surface distance as represented by the yellow–red colours in the topographic map (Fig. 4a, b). However, CT did not depict the articular cartilage shown as green–blue areas near the base of the femoral condyles, with a surface distance to the SLS model corresponding to that of the reported thickness for femoral hyaline cartilage (2–2.6 mm) [25, 26]. Furthermore, CT did not always depict the edge of osteophytic regions. This was not the case for 3D US, for which the colour map for the 3D US-SLS comparison revealed a colour distribution corresponding to a lower RMS surface distance, not only at the bone and articular cartilage surfaces, but at the osteophytic regions. This represents one important observation, considering that CT is well known for its ability to depict bone; however, it is limited on its ability to depict articular and periarticular soft tissues [27].

The evaluation of specimens 2 and 3 showed that the average RMS surface distance for 3D US-SLS was not statistically different than that of the CT-SLS comparison for each knee side. Additionally, coloured maps for such knee sides show similarity for all the surface distance values for both 3D US and CT at each of the osteophytic regions. Areas of maximum distances were noted particularly closer to the articular cartilage regions in the CT maps, while in the 3D US maps the colours appeared more evenly distributed. Together, these findings suggested similarity between 3D US and CT in bone depiction, yet superiority of 3D US at depicting osteophytes near areas of cartilage and fibrocartilage compared to CT for specimen 1, also observed for specimens 2 and 3.

Differences in osteophyte size may be correlated with their morphological composition. Kunz et al. and Al-attar et al. have reported cases of inaccurate osteophyte depiction from CT and have suggested that the accurate depiction of osteophytes from CT scans may be dependent on the stage of osteophyte development [5, 9], which ranges from more cartilaginous (early developing) to more calcified (more developed) [28]. CT parameters also play a role in the image acquisition quality and accuracy of osteophyte detection. Voltages between 100 and 120 kV are particularly favourable for smaller osteophyte depiction, whilst smaller slice thickness yields higher-resolution images [9, 29]. Consequently, the CT parameters for this study (0.625 mm slice thickness and 120 kV voltage exposure) were chosen based on such previous literature to produce the best quality images and osteophyte depiction possible. Observations during pre-screening and dissection revealed the medial side of specimen 1 to have small-to-medium sized osteophytes suggesting the possibility of early-stage osteoarthritis. Specimens 2 and 3 displayed large osteophytes, suggesting the possibility of later stage osteoarthritis. Altogether, these observations suggest that 3D US may be better at depicting smaller osteophytes (e.g., from specimen 1), but similar to CT for general osteophyte depiction, including depicting large osteophytes (e.g., from specimens 2 and 3).

There are limitations to the present study. Although this protocol was conducted on fresh frozen–thawed human cadavers, which is the closest experimental model to resemble a living patient [30], the number of donors and osteophyte regions were limited. Knee US is restricted by patellar shadowing and the lack of sufficient acoustic windows to visualize deep joint structures. Central osteophytes, however, tend to be associated with more severe changes of osteoarthritis than marginal ones [4, 31]. In this study, the scanning technique was chosen to allow visualization of osteophytes from significant portions of the medial and lateral joint spaces accessible for the US beam [6, 11, 12] which can be used as a predictor of degenerative changes within a joint. US is further associated with operator dependability, and images are notorious for poor signal-to-noise ratio and other artefacts that may compromise image resolution representing a challenge for the manual segmentation of 3D US volumes. A single operator conducted the US scanning and segmentations which were verified by senior experts in the field. Although studies have suggested that the evaluation of osteophytes with US can be learnt and performed by operators with limited experience [12, 13, 15], it remains important for future investigations to assess inter- and intra-observer reliability to validate osteophyte evaluations from US and the performance and agreement of the segmentations. It is also possible that despite utilising a validated atlas for pre-screening, some osteophytes were missed from the excluded knees. Future studies should be completed to validate the present findings in larger number of specimens to cover a broader spectrum of osteoarthritis progression, possibly allowing for significance or to help define a more informative tendency.

**Fig. 6:**
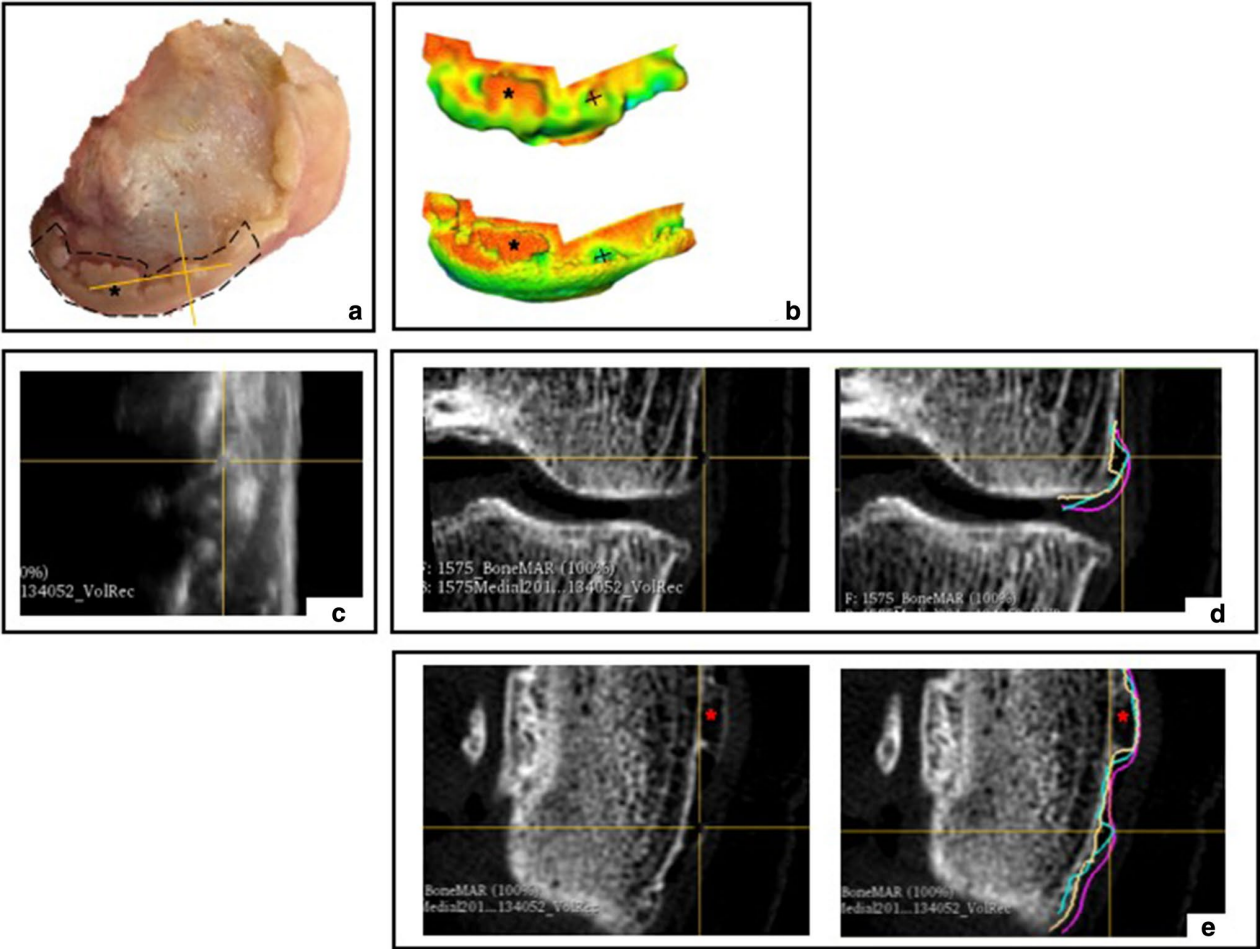
Observation of osteophyte depiction of specimen 2. **a** Medial side of specimen 2 with outlined regions of osteophytes. **b** Colour-coded topographic maps for 3D US-SLS comparison (top) and CT-SLS comparison (bottom); **c** coronal view of 3D US scan depicting anterior osteophyte; **d** coronal view of CT scan depicting anterior osteophyte with superimposed contours from the 3D US (blue), SLS (purple) and CT (yellow) models; **e** axial view of CT scan depicting anterior and posterior osteophytes with superimposed contours from the 3D US (blue) SLS (purple) models and CT (yellow) models. +  = anterior osteophyte; * = posterior osteophyte

The osteophyte areas circumscribed for each knee side were relatively large and did not account for the specific morphology of the osteophytes present. The medial side of specimen 2 was chosen for postliminary observations as it displayed a wide range of osteophyte size profiles (Fig. 6a). Smaller osteophytes, located anteriorly, appeared to have areas with slightly less error in the 3D US colour-coded maps than the CT coloured maps, whilst more prominent osteophytes located posteriorly, appeared to be well depicted in both modalities (Fig. 6b). The original image scans were visually compared to the 3D generated models of these specific osteophyte regions (Fig. 6c-e). Prominent and possibly well-developed and calcified osteophytes were depicted by both modalities. However, smaller osteophytes, possibly immature and cartilaginous, were not picked up by the CT scan, but were well detected by 3D US. Such findings could suggest that not all osteophytes are equal and that there are clear differences in depiction, yet the overall RMS surface distances were similar between 3D US and CT for this particular region. This may have been a result of averaging of the RMS surface distances along the knee sides, and thus may not accurately represent the heterogeneous nature of all osteophytes present. In future studies, averaging may be overcome by partitioning osteophyte regions according to size prior to obtaining the RMS surface distances, in order to investigate the correlation between differences in size and osteophyte depiction.

Overall, findings from this study suggest that 3D US can depict osteophytes with an average accuracy similar to that of CT, but the current study set-up cannot provide definitive proof. The accuracy of osteophyte depiction measurements, reported as RMS distance errors, was dependent on the accuracy of the registration between the models which may have been influenced by: the use of three different modalities, acquired at different spatio-temporal points, with differences in their reported resolutions, and incidental manual errors of the annotator during segmentation. Nonetheless, registration is often associated with a degree of uncertainty, and it remains a challenge to acquire submillimetre registration errors. Additional metrics should be considered in future studies to quantify osteophytes, e.g. diameter measurements in all directions; and to better characterize the surface match between the contours, e.g. Hausdorff distance.

The long-term objective is to apply such research protocol in a clinical setting. 3D US could represent a low-cost, dynamic and minimally invasive modality for capturing osteophytes, which can be incorporated into routine diagnostic evaluations and pre-operative planning. Automated segmentation, and 3D US to CT registration techniques [19, 21, 32–36] have been published to overcome the limitations of registration and manual segmentation which are not practical clinically. Advances in automatic segmentation algorithms include the ability to distinguish tissue features, such as cartilage thickness and bone contours, from noisy US images of the knee [35] and other anatomical regions such as the spine [36], hip [32] and pelvis [19]. Such research represents a promising avenue for the development of algorithms for the automatic localization osteophytes from US.

This study’s preliminary observations illustrate that differences in osteophyte depiction between 3D US and CT may be attributed to the heterogeneous morphology of osteophytes, and such findings will require further histomorphological evaluation to correlate the composition (cartilaginous vs calcified) and size (prominent vs small) of osteophytes with the depiction capabilities of each modality. Whilst 3D US represents important tool for first-line screening for osteoarthritis, further research needs to be conducted to determine the accuracy of 3D US in depicting osteophytes in the knee joint when compared to CT in vivo. Earlier work has demonstrated the feasibility of using SLS intraoperatively during total joint arthroplasties [37]. The three-way validation system presented in this study together with the RMS analyses could be applied at the sites of osteophytes in models derived from pre-operative 3D US and pre-operative CT, registered to intraoperative SLS, to quantify osteophyte depiction accuracy within a CAOS workflow. It is worth noting that CT is performed at a lower resolution in the clinic which may possibly compromise osteophyte depiction from CT to a greater extent in vivo. In addition to this, osteophyte depiction accuracy of 3D US should be validated against MRI, the golden standard for soft tissue imaging.

## Conclusion

Findings from this study illustrate that 3D US can depict osteophytes that appeared to be not always well depicted by CT, which may be attributed to the heterogeneous morphology of osteophytes. 3D US appeared to be sensitive to both the presence and the extent of osteophytes, as it was able to detect not only large and possibly calcified types of osteophytes but also less prominent possibly cartilaginous osteophytes which were not well depicted on CT. Together these findings suggest that US and 3D US could provide a more comprehensive depiction of osteophytes compared to CT. This investigation puts forward the value of 3D US for the screening and monitoring of osteoarthritis features such as osteophytes. Furthermore, it follows that a similar methodology could be used to investigate, in a clinical setting, the potential of 3D US as a viable pre-operative imaging modality.

## Data Availability

The authors confirm that the data supporting the findings of this study are available within the article and derived data supporting the findings are available from the corresponding author on request.
